# The increased level of COX-dependent arachidonic acid metabolism in blood platelets from secondary progressive multiple sclerosis patients

**DOI:** 10.1007/s11010-016-2770-6

**Published:** 2016-08-09

**Authors:** Agnieszka Morel, Elzbieta Miller, Michal Bijak, Joanna Saluk

**Affiliations:** 1 Faculty of Biology and Environmental Protection, Department of General Biochemistry, University of Lodz, Pomorska 141/143, 90-236 Lodz, Poland; 2 Department of Physical Medicine, Medical University of Lodz, Pl. Hallera 1, Lodz, Poland; 3 Neurorehabilitation Ward, III General Hospital in Lodz, Milionowa 14, Lodz, Poland

**Keywords:** Multiple sclerosis, Blood platelets, Arachidonic acid, Cyclooxygenase

## Abstract

Platelet activation is increasingly postulated as a possible component of the pathogenesis of multiple sclerosis (MS), especially due to the increased risk of cardiovascular events in MS. Arachidonic acid cascade metabolized by cyclooxygenase (COX) is a key pathway of platelet activation. The aim of our study was to investigate the COX-dependent arachidonic acid metabolic pathway in blood platelets from secondary progressive multiple sclerosis (SP MS) patients. The blood samples were obtained from 50 patients (man *n* = 22; female *n* = 28), suffering from SP MS, diagnosed according to the revised McDonald criteria. Platelet aggregation was measured in platelet-rich plasma after arachidonic acid stimulation. The level of COX activity and thromboxane B_2_ concentration were determined by ELISA method. Lipid peroxidation was assessed by measuring the level of malondialdehyde. The results were compared with a control group of healthy volunteers. We found that blood platelets obtained from SP MS patients were more sensitive to arachidonic acid and their response measured as platelet aggregation was stronger (about 14 %) relative to control. We also observed a significantly increased activity of COX (about 40 %) and synthesis of thromboxane B_2_ (about 113 %). The generation of malondialdehyde as a marker of lipid peroxidation was about 10 % higher in SP MS than in control. Cyclooxygenase-dependent arachidonic acid metabolism is significantly increased in blood platelets of patients with SP MS. Future clinical studies are required to recommend the use of low-dose aspirin, and possibly other COX inhibitors in the prevention of cardiovascular risk in MS.

## Introduction

Multiple sclerosis (MS) is a chronic neuroinflammatory and immune-mediated disease associated with the formation of central nervous system (CNS) inflammatory plaques as well as lesions exhibiting extensive demyelination, along with loss of oligodendrocytes, neurons, and axons [1]. MS is considered as a heterogeneous neurological disease with various pathophysiological mechanisms and multiple clinical course, but closely related to the damage of intracerebral blood vessels, mainly as a result of increased permeability of blood–brain barrier (BBB) as well vessel occlusion [2]. There exists four subtypes of MS: relapsing-remitting (RR), secondary progressive (SP), primary progressive (PP), and progressive-relapsing (PR). The most prevalent form of MS is RR MS, in which the disease fluctuates between periods of inflammation and demyelination, and remission. Ultimately, after several years of disease duration, RR MS in approximately 70 % of cases, converts into a SP MS in which patients suffer irreversible disability progression. The progressive phase of MS is believed to be secondary to neurodegenerative changes triggered by inflammation. In progressive MS, as in relapsing-remitting MS, active tissue injury is associated with inflammation, but the inflammatory response in SP MS occurs at least partly behind the blood–brain barrier [3]. PP MS is characterized by worsening neurologic function from the onset of symptoms, without relapses and remissions. PR MS is one of the rarest subtype of MS occurring in about 5 % of people with MS. In this subtype of MS a steadily worsening of the disease from the beginning and acute relapses are observed. The remissions do not occur in patients with PR MS [4].

Epidemiological studies confirm an increased risk of cardiovascular disease in MS, especially ischemic stroke and myocardial infarction that is acute events directly associated with abnormal platelet functions and their prothrombotic activity [5, 6]. It is thought that blood platelets play a crucial role in neurodegenerative processes, in which an excessive activation of platelets are observed [7]. A various bioactive compounds stored in platelet α-granules and released upon their activation may affect the permeability of BBB, and be crucial for the infiltration of T-lymphocytes, responsible for the dissemination of new inflammatory lesions in the CNS [8]. Our previous findings suggest that increased platelet activation may be an important cause of hemostatic disorders occurring in the progressive stage of MS (SP MS). Platelets are most likely important determinants in the pathogenesis of MS and actively participate in oxidative stress existing in SP MS [9]. Inhibition of platelet activation can provide measurable benefits in suppressing the disease process in MS.

Upon platelet activation the signal transduction leads to mobilization of calcium and increases its intracellular concentration, resulting in phospholipases activation. These enzymes hydrolyze phospholipids of cell membrane, releasing e.g., the arachidonic acid (AA), which is a precursor of essential bioactive eicosanoids. AA is enzymatically transformed by the cyclooxygenase (COX) to intermediate products: prostaglandins and thromboxane A_2_ (TXA_2_), and then calcium is removed from intracellular storage sites [10, 11]. TXA_2_ is a potent blood platelet activator acting as proaggregatory and vasoconstrictor mediator, which plays a pivotal role in the growth and stabilization of a coronary thrombus [11]. This compound is formed in response to the local stimuli and it exerts its activating effect within a short distance of its biosynthesis. COX activation is associated with prothrombotic platelet activity and the production of proinflammatory eicosanoids. AA cascade metabolized by COX is a key pathway of platelet activation. The addition of AA to platelet-rich plasma in vitro, results in a burst of oxygen consumption, TXA_2_ formation, and platelet aggregation [12]. The major clinical indication for antiplatelet pharmacotherapy is the prevention of arterial thrombosis. Clinically used agents are based on interrupting specific sites in the sequence of platelet activation. The results of clinical studies have shown that intake of the antiplatelet agents as aspirin or different aspirin-like COX inhibitors, at low doses reduces the incidence of cardiovascular events [13]. Low-dose aspirin supplementation reduced the risk of serious cardiovascular events by 12 % and nonfatal myocardial infarction by 18 % [14].

COX is an enzyme present in many types of cells. COX-1 is constitutive cyclooxygenase involved both in the maintenance of homeostasis in normal conditions and in the early stages of an inflammatory reaction. The inducible cyclooxygenase (COX-2) is involved in the later stages of an inflammatory reaction when it starts the development of the immune response. COX-1 is constitutively expressed in blood platelets, as well as in neurons, astrocytes, and microglial cells [15]. This enzyme plays a key role in the conversion of AA to essential cell-signaling eicosanoids which is accompanied by the production of reactive oxygen species (ROS) [16]. It is believed that oxidative stress might be responsible for brain inflammatory disorders causing deleterious effects during CNS pathogenesis. What is more, oxidative stress can activate several intracellular signaling cascades that can have deleterious effects on the cellular homeostasis [17].

Dysfunction of the mitochondrial system is thought to play a major role in the mechanism of progression of various neurodegenerative disorders, including demyelinating disorders in MS. Although the weight of evidence demonstrates that, while the development of pathology in the early stages of MS is largely driven by inflammation, mitochondrial dysfunction appears to have a critical role in the progression of this disease [18]. Mitochondrial abnormalities in MS is presented by altered structure and distribution coupled with a wide array of molecular and biochemical abnormalities [19–21]. In the studies conducted by Mao et al. on the role of mitochondrial abnormalities in patients with MS and EAE mouse models, the authors revealed that mitochondrial DNA defects, abnormal mitochondrial gene expression, defective mitochondrial enzyme activities, deficient mitochondrial DNA repair activity, and mitochondrial dysfunction are involved in the development and progression of MS. They also explain that mitochondrial abnormalities and mitochondrial energy failure can impact other cellular pathways, including increased demyelination and inflammation in neurons and tissues that are affected by MS [22]. Deficient mitochondrial metabolism leads to the increased production of ROS that can wreak havoc in the cell and which is detrimental to neurons and glia [22, 23]. Therefore, mitochondria dysfunction is closely related to the mechanism of neuroinflammation. In turn, neuroinflammation is increasingly recognized to produce mitochondrial failure, which then contributes to further neuronal injury and degeneration [23].

The main aim of our studies was to determine the pathophysiological mechanisms of blood platelet activation related to COX-1 activation in SP MS patients. We quantified platelet activation by measuring the level of COX-1 activity and TXB_2_ formation in patients diagnosed with SP MS using ELISA methods. TXA_2_ as a main product of AA metabolism itself is not stable enough for monitoring purpose. TXA_2_ is rapidly metabolized into more stable metabolite—TXB_2_ that can be detected in the serum. Moreover, we estimated the reactivity of blood platelets assessing their aggregating response to AA—as their physiological agonist. ROS generation and lipid peroxidation were determined by the level of thiobarbituric acid reactive substances (TBARS) expressed mainly as malondialdehyde (MDA) as the most common marker of lipid peroxidation.

## Materials and methods

### Demographic and clinical characteristics

The blood samples were delivered from Neurological Rehabilitation Division III General Hospital in Lodz, Poland. Blood was collected from 50 patients (man *n* = 22; female *n* = 28) suffering from SP MS. Patients were observed for 1 year before and were diagnosed according to the revised McDonald criteria. SP MS was ascertained as defined by Lubin et al. [24]. Clinical parameters in patients with SP MS: mean age −48.2 ± 15.2 years, mean disease duration −14.3 ± 8.3 years, BMI −21.1 ± 9.7. The patients were under Neurorehabilitation Ward control for 3 months and in that time they didn’t receive any immunostimulators, immunomodulators, hormones, minerals, vitamins, or any other substitutions with antioxidative effect. Treatment with immunomodulating therapies was not used in this progressive stage of MS for almost 1 year. This kind of inclusion criteria allow us to avoid interference of effects of these drugs on oxidative stress parameters.

Control human blood samples were delivered from fifty healthy volunteers, not taking any medications, who have never been diagnosed with MS or other chronic disease and without any neurological, hormonal illness, and any chronic inflammatory. The control group and patients with SP MS matched by the age and sex.

The protocol and all procedures were done according to the Helsinki Declaration and were approved by Ethics Committee of the Faculty of Biology and Environmental Protection of University of Lodz, Poland No. 5/KBBN-UŁ/II/2013.

### Isolation of platelet-rich plasma and blood platelets

The human blood samples (from control group and patients) were collected into CPDA-1 (citrate phosphate dextrose adenine-1), taken from a peripheral vein between 8 and 9 am in fasting status and stored using the same protocol. The differential centrifugation of blood was used for isolating platelet-rich plasma (PRP) for 10 min at 1500 rpm. To get blood platelets, PRP was centrifuged for 15 min at 2500 rpm. PRP was used to measure aggregation. The isolated and purified platelets were used to determine the level of TBARS and activity of COX. The final concentration of platelet suspension was approximately 4 × 10^8^/ml. The number of platelets was counted by the photometric method according to Walkowiak et al. [25]. Platelets were suspended in the modified Tyrode’s (Ca^2+^/Mg^2+^) free buffer (127 mM NaCl, 2.7 mM KCl, 0.5 mM NaH_2_PO_4_, 12 mM NaHCO_3_, 5 mM HEPES, 5.6 mM glucose, pH 7.4). Samples of blood platelets for the determination of COX fluorescent activity were dissolved in lysis buffer 1:1 (7 M Urea, 2 M Thiourea, 4 % CHAPS, 30 mM Tris).

### Platelet aggregation

The platelet aggregation was measured turbidimetrically in PRP using the optical Chrono-Log aggregometer (Chrono-Log, Havertown, PA). After preincubation of PRP (3 × 10^8^ platelets/ml, at 37 °C for 5 min) physiological agonist was added—free AA (1 mM). The aggregation was measured with stirring by the duration of 10 min. The results are presented as a percentage of the aggregation. The maximal aggregation (100 %) was defined as the light transmission observed in PPP (platelet-poor plasma).

### Determining the COX-1 fluorescent activity

In platelet lysates obtained from patients with SP MS and healthy volunteers the level of COX-1 activity was determined by the fluorescence-based method, using COX Fluorescent Activity Assay Kit (Cayman Chemicals). The oxygenase activity of COX is responsible for the conversion of arachidonic acid to a hydroperoxy-endoperoxide (PGG_2_). In this assay, the reaction between PGG_2_ and ADHP (10-acetyl-3,7-dihydroxyphenoxazine) produces the highly fluorescent compound resorufin that can be analyzed using an excitation wavelength of 530–540 nm and an emission wavelength of 585–595 nm. The results are presented in nmol/min/ml.

### Determining the level of TXB_2_ by competitive ELISA assay

The human blood samples (from control group and patients) were collected into serum tubes with coagulation activator (without anticoagulant). To quantify the level of TXB_2_ in serum obtained from SP MS patients and healthy control, the Thromboxane B_2_ Express ELISA Kit—Monoclonal (Cayman Chemicals) was used. The total level of TXB_2_ in all samples was expressed in pg/ml.

### Thiobarbituric acid reactive substances estimation

Samples of blood platelet suspended in the modified Tyrode’s buffer were mixed with an equal volume of 15 % (w/v) cold trichloroacetic acid in 0.25 M HCl and with an equal volume of 0.37 % (w/v) thiobarbituric acid in 0.25 M HCl. All samples were immersed in a boiling water bath for 10 min. After cooling samples were centrifuged and then the absorbance at 535 nm was measured. The results were expressed as nmoles of MDA per ml of platelet suspension, as we described previously [26].

### Statistical analysis

The statistical analysis was performed using StatsDirect statistical software V. 2.7.2. All values were expressed as a mean ± SD. To analyze the normality of the distribution of results, the Shapiro–Wilk test was used. The significance of differences between the values were analyzed depending on the normality by unpaired *t*-student (for data with normal distribution) or U-Mann–Whitney (for data with abnormal distribution) tests. Spearman’s rank correlation was used in correlation analysis between markers of platelet activation (COX-1, TXB_2_) and clinical parameter of physical disability—EDSS scores. A level of *p* < 0.05 was accepted as statistically significant.

## Results

**Fig. 1:**
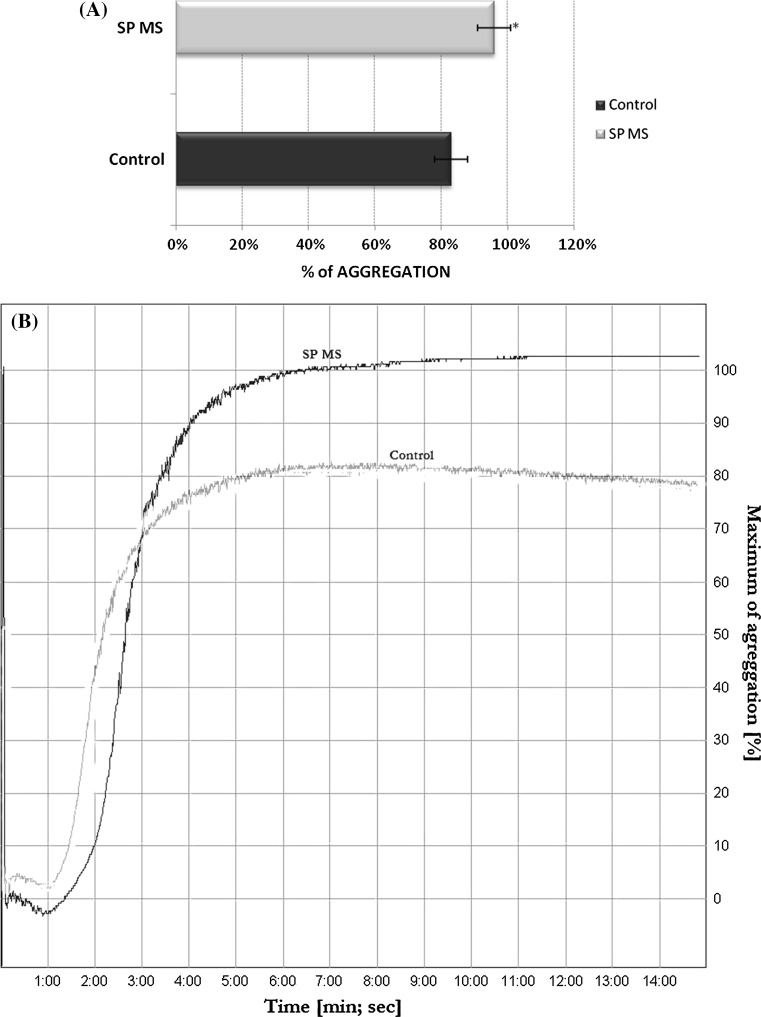
Blood platelet aggregation induced by arachidonic acid in platelet-rich plasma. Data represent mean ± SD, *n* = 50. Statistical analysis was performed using Mann–Whitney U-test *p < 0.0001 SP MS versus control (a). Typical curve of platelet aggregation in PRP after stimulation of platelets with AA (Chrono-Log aggregometer, Havertown, PA) (b)

Measurement of platelet aggregation was performed on each sample. To ensure full platelet activation a physiological stimulus—AA (1 mM) was added. The amounts of agonist was constant and sufficient to activate platelets in whole samples. The activation of platelets in PRP upon AA stimulation resulted in the statistically significant increase in the platelet aggregation (*p* < 0.0001) in SP MS patients versus control group (Fig. 1a, b). The level of platelet aggregation increased from 87 % for healthy control to 99 % for SP MS patients (growth of 14 % of control—when the value of the control was taken as 100 %) (Fig. 1a).

**Fig. 2:**
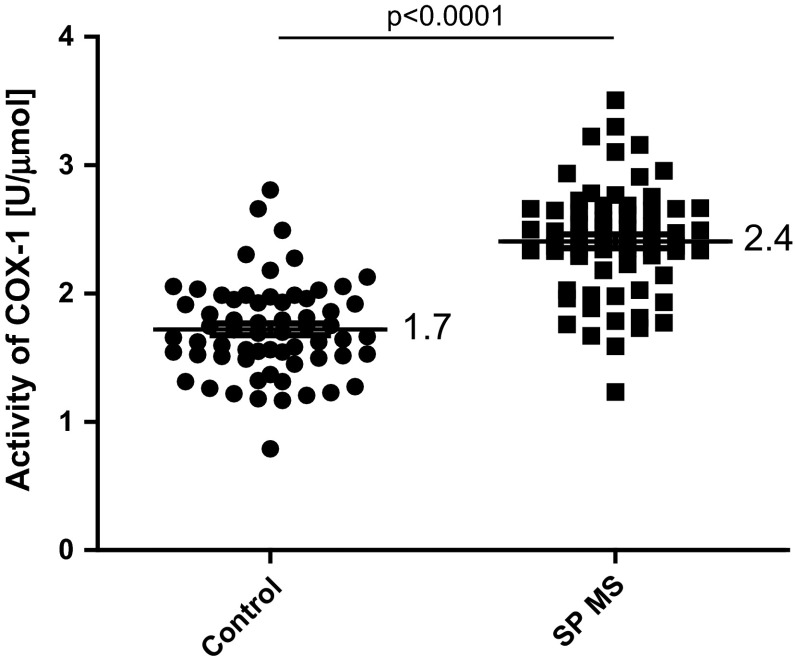
The level of oxygenase activity of COX-1 in platelet lysates from SP MS patients and healthy controls. Data represent mean ± SD, *n* = 50. Statistical analysis was performed using unpaired *t*-test *p < 0.0001 SP MS versus control

**Fig. 3:**
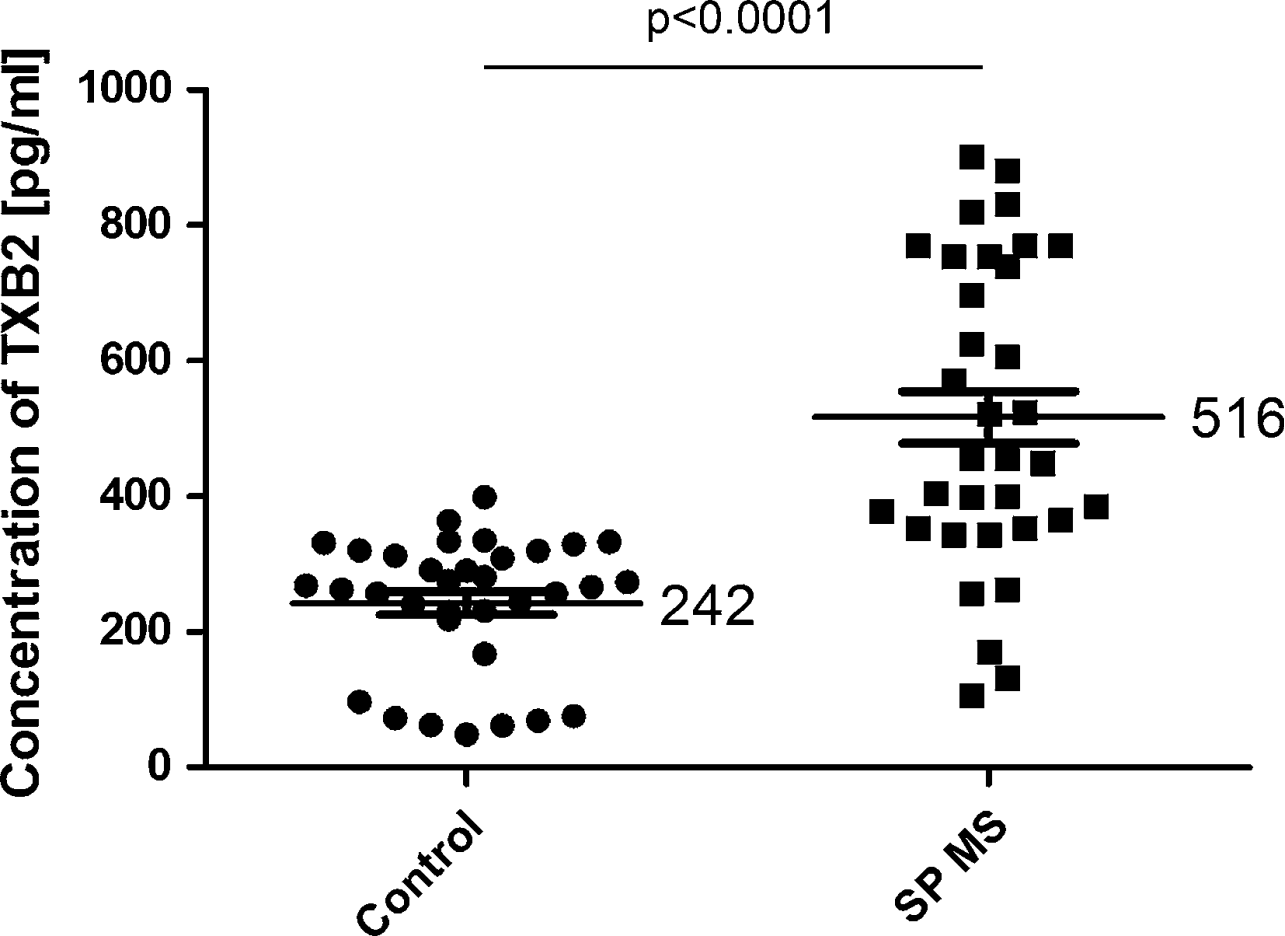
The concentration of TXB_2_ in serum obtained from SP MS patients and healthy controls. Data represent mean ± SD, *n* = 34. Statistical analysis was performed using unpaired *t*-test *p < 0.0001 SP MS versus control

**Fig. 4:**
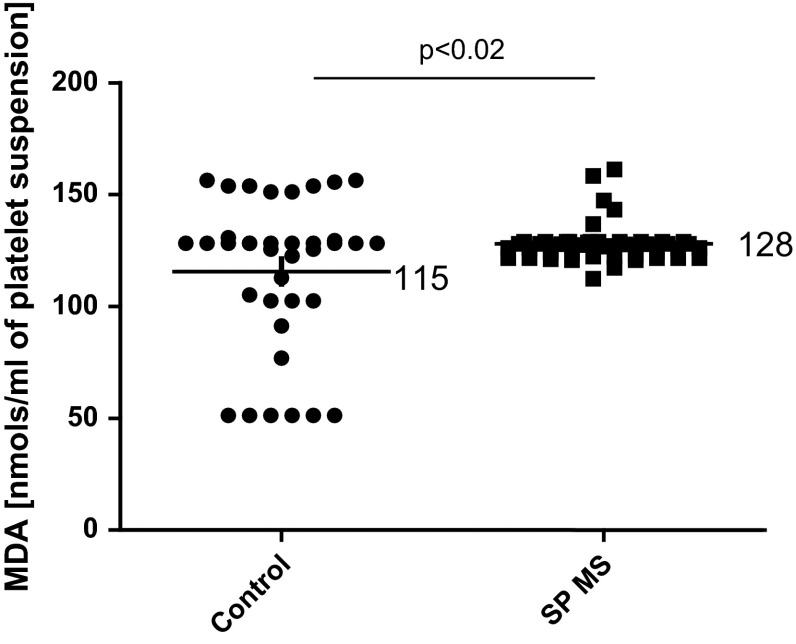
The level of TBARS concentration measured in platelet samples obtained from SP MS patients and healthy controls. Data represent mean ± SD and are expressed as nmol of MDA/ml of platelet suspension, *n* = 35. Statistical analysis was performed using unpaired *t*-test *p < 0.02 SP MS versus control

Moreover, our results clearly indicated the statistically significant increase in basal platelet activation state in patients with SP MS compared with healthy individuals. The level of oxygenase activity of COX-1 measured in platelets by ELISA method, was higher over 40 % (*p* < 0.0001) in SP MS patients than in control group (Fig. 2). Similarly, we observed the statistically significant increase in the production of thromboxane (*p* < 0.0001). The concentration of TXB_2_, determined by ELISA method, in blood platelets derived from SP MS patients was more than twofold higher than in controls (Fig. 3). Our results also revealed that in blood platelets from patients with SP MS the synthesis of AA metabolites, measured as the amount of TBARS, and expressed as nmoles of MDA per ml of platelet suspension, was increased by approximately 10 % (*p* < 0.02) compared with healthy subjects (Fig. 4).

**Fig. 5:**
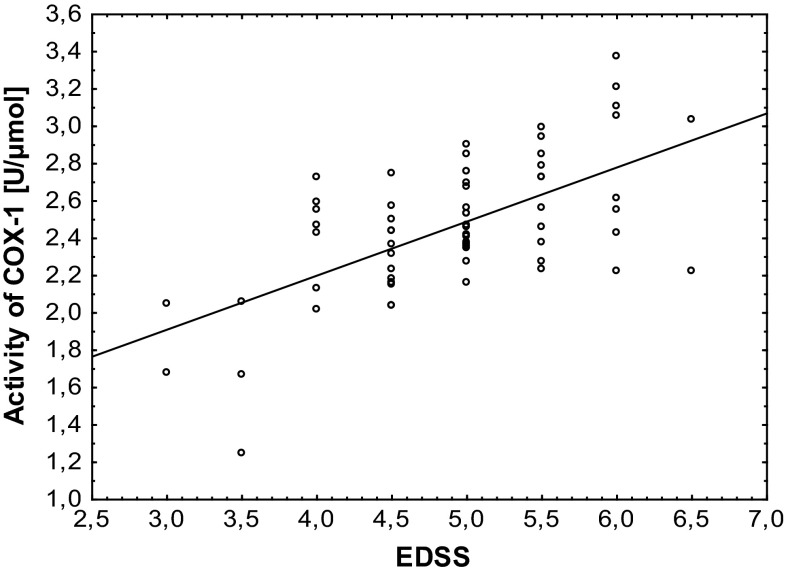
Regression plot of COX-1 activity in platelets obtained from SP MS patients and EDSS score

**Fig. 6:**
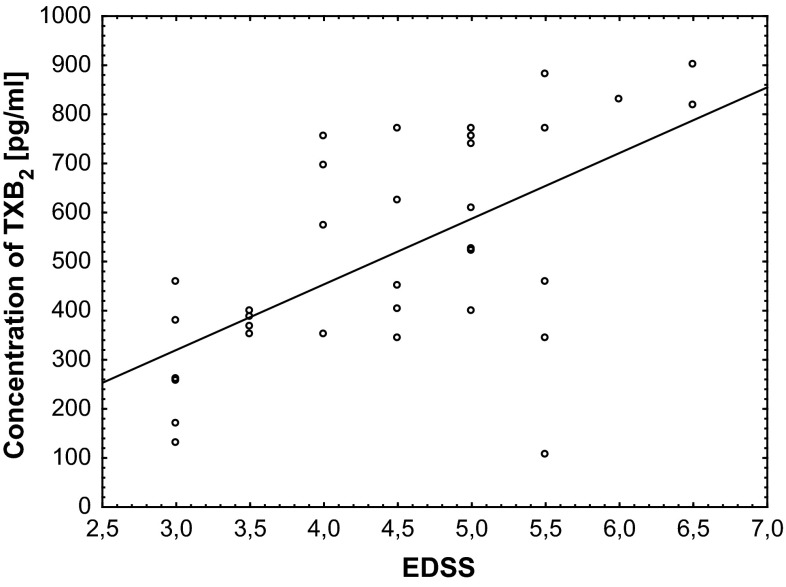
Regression plot of TXB_2_ level in platelets obtained from SP MS patients and EDSS score

We also established the positive correlation between the level of COX-1 activity (Fig. 5; Table 1), the level of TXB_2_ (Fig. 6; Table 1), and EDSS score.

**Table 1:** Correlation coefficient values obtained for level of COX-1 activity, level of TXB_2_, and EDSS score

	COX-1	TXB_2_
EDSS		
Spearman’s rank correlation (*ρ*)	0.6223	0.6155
Probability for correlation	*p* = 0.00001	*p* = 0.0001

Correlation was analyzed using Spearman’s rank correlation method. Table consists Spearman’s rank correlation coefficient (*ρ*) and probability for correlation (*p*)

## Discussion

Some studies suggest that patients with multiple sclerosis have an increased risk for cardiovascular events, for example, venous thromboembolism, ischemic strokes, and myocardial infarction. These clinical observations suggest that there is a possibility that aspirin (or other aspirin-like drugs) could diminish this increased risk in this population [27]. Aspirin (acetylsalicylic acid) is a well known and popular COX inhibitor, and it is often used for the secondary prevention of cardiovascular events in patients at elevated risk. This drug has the potential to limit some components of inflammation (production of proinflammatory cytokines and ROS by microglia) and may also positively impact other disease process in multiple sclerosis, for example, it is likely to facilitate remyelination efforts by promoting the proliferation and differentiation of oligodendrocyte precursors [28].

In Modi et al. [29] research on the role of ASA in MS patients, the authors clearly support the conclusion that aspirin is capable of up-regulating ciliary neurotrophic factor (CNTF) in mouse in vitro and human astrocytes in vitro. Aspirin may ameliorate demyelination. Aspirin-induced astroglial CNTF was also functionally active because the supernatants of aspirin-treated wild type (WT), but not CNTF^−/−^, astrocytes-supported oligodendroglial growth as evidenced by increased expression of proteolipid protein (PLP) and myelin oligodendrocyte glycoprotein (MOG). Furthermore, aspirin-induced CNTF-protected oligodendroglia from TNF-α-induced apoptosis and cell death. The authors have demonstrated that aspirin, a widely used analgesic, up-regulates CNTF via PKA-mediated activation of cAMP response element-binding protein (CREB) and that aspirin-treated astroglial supernatant protects oligodendroglia from inflammatory insult via CNTF. Although the in vitro situation of mouse astrocytes and oligodendrocytes in culture and its treatment with aspirin may not truly resemble the in vivo situation of these cells in the brain of MS patients, these results highlight an undiscovered property of aspirin and indicate that this drug may be used for therapeutic intervention in MS and other demyelinating conditions as primary or adjunct therapy.

Acetylsalicylic acid has been shown to be effective in reducing cardiovascular disease conditions associated with thrombosis and an increased blood platelet activation. The inhibitory action of aspirin on platelet function is due to acetylation of the platelet cyclooxygenase at the functionally important amino acid Ser-529 of COX-1 and Ser-516 of COX-2. This prevents the access of arachidonic acid (COX substrate) to the enzyme catalytic site at Tyr-385 and results in an irreversible inhibition of platelet formation of prothrombotic and proinflammatory mediators [30]. COX is a membrane-bound glycoprotein which possesses two distinct enzymatic activities: oxygenase and peroxidase. The COX pathway is substrate limited and is strictly dependent on the release of arachidonic acid from the phospholipid membrane, because only free, unesterified AA is an oxygenase substrate [31]. AA is a prominent fatty acid in platelet phospholipids and phosphatidylcholine, phosphatidylethanolamine, and phosphatidylinositol contain even 80 % of arachidonate. Enzymes participate in AA release are phospholipase A_2_ and phospholipase C. Free arachidonic acid is the substrate for the synthesis of biologically active eicosanoids (prostaglandins and thromboxanes) [10]. The initial step in their synthesis is oxygenation of AA at C-11 by COX. The oxygenase activity catalyses the incorporation of two oxygen molecules to one molecule of arachidonate, creating a prostaglandin PGG_2_. After oxidation, the enzyme is irreversibly inactivated by self-destruction. While peroxidase catalysis results in the reduction of 15-OOH group of PGG_2_ to 15-OH, and formation of PGH_2_, which is a substrate for thromboxane synthesis [32]. TXA_2_ is a potent stimulus to platelet aggregation, as well a major second mediator in the platelet response to exogenous agonists. TXA_2_ is an unstable molecule with a short half-life about 30 s., after which it passes to the stable but inactive compound TXB_2_ [33]. Participation of blood platelets in both physiological processes and pathological conditions is dependent on their activation. The resting platelets do not contain free arachidonate and therefore there is no pathway of platelet AA metabolism. Since both the enzymes (phospholipases C and A_2_) involved in hydrolysis of membrane phospholipids, are calcium dependent, release of free AA is evidently associated with an intracellular Ca^2+^ flux [34].

It is thought that platelet activation may be an epiphenomenon consequent to the disease processes in MS, probably secondary to endothelial injury, which causes the exposure of platelets to a variety of stimuli. Some reports emphasize the importance of platelet activation in MS [35]. Our previous studies also clearly demonstrated that blood platelets are chronically activated in the circulation of SP MS patients [36]. However, there are numerous studies describing the physiology of platelets in the relapsing-remitting phase of MS (RR MS)—the first stage of disease and the most common disease course, but only very few studies on the role of platelets in SP MS. Among the existing studies, the differences in platelet activation parameters between RR MS and SP MS exist. Higher level of sP-selectin and other markers of platelet activation [37], as well as an increase of PAF activity [38] were observed in RR MS compared to secondary progressive MS. In Sáenz-Cuesta et al. studies, the authors observed the higher level of the platelet-derived microparticles (MPs) in patients with RR MS than in SP MS [39].

Based on the fact that each clinical phase of MS should be considered as a distinct disease entity [40], we focused our attention on the alterations of platelets in progressive phase (SP MS) characterized by irreversible disability progression [41]. In our previous studies the elevated activation of platelets and an increased aggregation in response to typical physiological agonists (ADP, collagen) have been observed in SM patients [9, 36]. Activation of COX pathway is recognized to participate in intraplatelet events associated with aggregation. After platelet activation by thrombin, collagen or ADP, synthesis of TXA_2_ is responsible for platelet aggregation. Synthesized eicosanoids are not stores, and therefore, the action of TXA_2_ indicates the activation of COX [42]. COX-dependent metabolism of AA is a key pathway of platelet activation. In the present study we focused on the assessment of changes of COX- dependent arachidonic acid metabolism in blood platelets from SP MS patients. Our results demonstrate that oxygenase activity of COX in platelets from SP MS patients is significantly elevated (about 40 %) compared to platelets from healthy control (Fig. 2). Also peroxidase activity of COX is significantly higher in SP MS patients, which results in pronounced, more than twofold increase in the concentration of TXB_2_ (Fig. 3). In these studies, for the first time, we have shown the positive correlations between the level of COX-1 activity (Fig. 5; Table 1) and the level of TXB_2_ (Fig. 6; Table 1) in blood platelets obtained from SP MS patients with the clinical parameter EDSS score. It clearly indicates that the platelet activation is associated with the physical disability of disease. Our earlier and current findings confirm the abnormalities in platelet hemostatic function and indicate the elevated platelet activation. We postulate that it is probably related to a direct action of thromboxane, which modifies the response of platelets to exogenous agonists as well as alone stimulates platelet activation. Platelet surface receptor for TXA_2_ (TP) is a member of the seven-transmembrane G protein-coupled receptor superfamily. Binding of ligand to TP results in both autocrine and paracrine action of TXA_2_ released from blood platelets. In TP signaling pathway two G proteins are involved: G_q_ and G_12/13_. Stimulation of G_q_ activates phospholipase C, resulting in the accumulation of inositol 1,4,5-trisphosphate causing intraplatelet calcium mobilization, and diacylglycerol formation, which induces activation of protein kinase C. In platelets, this signaling results in platelet shape change, aggregation, and secretion. Stimulation of G_12/13_ activates the Rho/Rho kinase pathway and subsequent myosin light chain phosphorylation, which is responsible for TP-mediated platelet shape change [33].

Our present studies were designed to demonstrate the platelet aggregation induced by extracellular AA. The exogenous arachidonic acid is able to cause platelet irreversible aggregation, because it may be rapidly incorporated into membrane phospholipids, primarily phosphatidylcholine and phosphatidylinositol, by arachidonoyl-coenzyme A synthetase. This aggregation response induced by exogenous AA depends on COX activity, and is inhibited by aspirin [43]. In our experiments the platelet aggregation measured in PRP derived from SP MS was higher by about 17 % than in healthy control (Fig. 1). Our previous and current results indicate that platelets from SP MS patients are much more sensitive to physiological agonists and their responsiveness is stronger than platelets from healthy subjects.

It is well known, that platelet arachidonate metabolism generates a great amount of ROS. After platelet activation, COX rapidly metabolizes free AA to PGH_2_ causing the burst of oxygen consumption [44]. ROS generation induces lipid peroxidation that is responsible for changes in the fluidity of membranes, their permeability, and exposition of the receptors, and results in changes in signal transduction [45]. Our studies confirm the elevated level (by about 10 %) of lipid peroxidation in platelets obtained from SP MS patients (Fig. 4).

ROS are implicated in the regulation of platelet function and may be produced as second messengers, in the receptor-mediated signaling pathways during platelet activation [46]. Many pathogenic processes, including blood platelet hyperactivity, may be initiated by injury action of ROS; therefore, blood platelets link the processes of hemostasis, thrombosis, and inflammation. The superoxide radicals may interact with iron (accumulated in the brain in SP MS) and form reactive hydroxyl radicals that attack polyunsaturated fatty acids, leading to lipid peroxidation and demyelination [9]. In blood platelets, the main target of ROS/RNS action are proteins [4, 47]. They can be modulated in direct and indirect ways. Direct modifications involve: nitration, carbonylation, and disulfide bond formation [48, 49]. Exposure of platelet proteins to ROS/RNS can alter every level of protein structure from primary to quaternary, causing major physical changes in protein structure [4, 47, 48]. The oxidative/nitrative damage of platelet proteins leads to the peptide backbone cleavage, cross-linking, and/or modifications of the side chain of every amino acid [50]. The increased protein aggregates, in turn overwhelms the degradation systems which results in a self-perpetuating cycle and further oxidative stress. The great number of protein injury is irreparable, and oxidative/nitrative changes of protein structure have functional consequences, such as an inhibition of enzymatic activities, a misfolding, an increased ability of proteins for aggregation and proteolysis, and an altered immunogenicity [51]. Individual proteins can display different susceptibilities to oxidative attack, linked to the distribution of sulfhydryl (SH) groups [52]. ONOO^−^ is a highly reactive compound that modify tyrosine residues resulting in protein nitration, expressed as a 3-NT [53–55]. Tyrosine nitration inhibits the activity of manganese superoxide dismutase and phosphorylation process which leads to the redox imbalance and disturbances in signaling pathways [56].

The main function of blood platelets is their hemostatic contribution, but evidences show that processes of platelet activation may be sometimes a critical link between hemostasis and development of inflammation. Platelets are activated in MS and have been implicated in contributing to MS pathogenesis, such as by promoting inflammation [26, 35]. Our results demonstrate, that the activation of platelets from SP MS patients is significantly raised compared to platelets from healthy subjects, and observed differences are directly related to the COX activation. COX may be inhibited by aspirin-like drugs, and inhibition is limited to the oxygenase activity, while peroxidase action is intact [57, 58]. Aspirin is the most well-tolerated and effective antithrombotic agent. Aspirin is an approximately 150- to 200-fold more potent inhibitor of the constitutive isoform of platelet enzyme responsible for TXA_2_ synthesis, than the inducible isoform which is expressed by inflammatory mediators. This explains the different dosage requirements of aspirin as an antithrombotic (COX-1) and an antiinflammatory drug (COX-2), respectively [27].

Because, as we have shown in the work, increased platelet activity and their high reactivity in the circulation of SP MS patients is associated with elevated activity of COX, preventive use of safe doses of aspirin could reduce the number of cardiovascular events in these patients. The role of new, more specific antiplatelet drugs may also hold future promise in reducing dimension of platelet activation, and production of ROS and proinflammatory mediators.
